# Investigating the Potential for Reducing Sodium Bicarbonate in the Nutrient Medium for Cultivation of *Limnospira platensis* at Elevated CO_2_ Concentration

**DOI:** 10.3390/plants15213425

**Published:** 2026-11-04

**Authors:** Maksim A. Kravets, Elizaveta M. Kovalenko, Marina E. Vavilkina, Anatoly V. Grigorenko, Mikhail S. Vlaskin

**Affiliations:** Joint Institute for High Temperatures of the Russian Academy of Sciences, 125412 Moscow, Russia; kravets.ma@phystech.edu (M.A.K.); lizloom@mail.ru (E.M.K.); vavilkina-marina@rambler.ru (M.E.V.); presley1@mail.ru (A.V.G.)

**Keywords:** *Limnospira platensis*, spirulina, sodium bicarbonate, Zarrouk medium, elevated CO_2_, carbonate–bicarbonate equilibrium, pH, photobioreactor

## Abstract

Sodium bicarbonate (NaHCO_3_) in Zarrouk medium serves as both the main carbon source and an alkaline buffer, and it accounts for a large share of the cost of spirulina cultivation. This study assessed whether the NaHCO_3_ concentration in the medium can be reduced, down to complete elimination, for *Limnospira platensis* grown at elevated CO_2_ compared with atmospheric CO_2_. Two sequential experiments (12 days with CO_2_ enrichment to a 3% setpoint; up to day 8 at atmospheric CO_2_, by which time the cultures had died) were carried out in a hermetic gas chamber with 10 L column photobioreactors, using a strain adapted to 3% CO_2_. The experiments differed only in the chamber atmosphere (3% CO_2_ and atmospheric ~0.04% CO_2_), the CO_2_ concentration in the enriched chamber being raised to ≈3% after each sampling and declining to ≈0.5% by the next. In each, eight NaHCO_3_ concentrations (0–12 g/L, i.e., 0–100% of the reference level of 12 g/L, which is 71% of the standard 16.8 g/L in Zarrouk medium) were tested in triplicate at 3% CO_2_ and in duplicate at atmospheric CO_2_. Biomass, pH, carbonate and bicarbonate ions, nitrate and phosphate consumption, and trichome viability were monitored. At 3% CO_2_, the culture grew at all NaHCO_3_ concentrations, including medium without added NaHCO_3_, reaching 3.59–4.11 g/L of biomass by day 12 (7–8% less than in the 12 g/L control at 0 and 0.6 g/L). The pH remained within 8.40–9.40, and the proportion of live trichomes was 92.1–96.3%; trichome viability did not differ significantly between variants, whereas pH did. Under atmospheric CO_2_ (CO_2_-adapted inoculum transferred directly to air), the apparent pH rose to 12.75, bicarbonate was depleted, and the culture died within 2 days at 0–1.2 g/L NaHCO_3_ and by day 8 at 3–12 g/L, with no live trichomes remaining by day 6. These results indicate that, with CO_2_ enrichment of the chamber atmosphere (3% setpoint, ≈1.4% on average), added NaHCO_3_ is not required for growth over 12 days: the carbon fixed in the biomass was supplied from the gas phase, and the bicarbonate pool was formed in situ with alkalinity derived mainly from nitrate assimilation. This suggests a possible way to reduce medium costs in spirulina production, which has yet to be confirmed by a CO_2_ supply and cost analysis.

## 1. Introduction

Spirulina is a cyanobacterium of the species *Limnospira platensis* that is widely cultivated to obtain biomass rich in protein (60–70% of dry weight), vitamins, minerals, essential fatty acids, and other valuable compounds [1,2]. The standard medium for its cultivation remains Zarrouk medium, in which sodium bicarbonate (NaHCO_3_) serves simultaneously as the main carbon source and an alkaline buffer [3]. The high NaHCO_3_ concentration in the classical recipe (16.8 g/L) provides carbon nutrition for the culture and maintains the pH in an alkaline range favorable for growth; however, it is the cost of bicarbonate that makes the largest contribution to the cost of the nutrient medium—one of the main factors limiting the scaling-up of industrial spirulina biomass production [4]. Along with Zarrouk medium, other formulations are also used for spirulina cultivation (BG-11, BBM, FM-II, Arnon’s medium); however, Zarrouk medium and its modifications remain the most widely used [4,5,6,7], while reducing the NaHCO_3_ content is one of the most promising ways to lower cultivation costs [8].

For photosynthesis, spirulina has access to two forms of inorganic carbon—dissolved CO_2_ and bicarbonate ions HCO_3_^−^, the ratio of which in the medium is determined by pH [9]. The pH range optimal for spirulina growth (8.5–10.5) [10] shifts the carbonate equilibrium toward HCO_3_^−^, which is the preferred and more readily available carbon source compared with dissolved CO_2_ [11]. This is why the high NaHCO_3_ concentration in Zarrouk medium provides not only carbon nutrition but also alkaline buffering. When gaseous CO_2_ is sparged, it partially dissolves and, if the buffering capacity of the medium is insufficient, causes acidification [12]; at the same time, a significant fraction of CO_2_ is lost during aeration before it can dissolve [13]. Thus, replacing bicarbonate with gaseous CO_2_ is physicochemically feasible but requires maintaining the pH and minimizing gas losses.

A number of studies in the literature demonstrate that it is in principle possible to replace expensive NaHCO_3_ in Zarrouk medium with carbon dioxide; however, quantitative estimates of the permissible reduction and its effect on growth rate differ substantially between studies. Thus, in [14], NaHCO_3_ was completely replaced with a NaOH solution and CO_2_, which reduced the cost of the medium by 34.3% without compromising biomass. In [15], spirulina was grown on Zarrouk medium entirely without NaHCO_3_, using only 0.5% CO_2_ as the carbon source. In [16], by contrast, the maximum biomass gain was obtained not with complete exclusion of bicarbonate but at a concentration of 8.4 g/L—half the standard value—which indicates the existence of an optimum rather than a monotonic dependence. Previous studies have demonstrated the feasibility of cultivating *Spirulina platensis* in reduced-cost media, highlighting the potential to lower cultivation costs while maintaining biomass production [8]. At the same time, several studies point to the limitations of using gaseous CO_2_: at high concentrations (9 vol%), mass death of *Limnospira platensis* cells and a decrease in the lipid and protein content of the biomass are observed [17], and the specific growth rate in cultivation with 10% CO_2_ is significantly lower than without CO_2_ addition [18]. The situation is aggravated by the low efficiency of sparging-based gas exchange: the fraction of CO_2_ absorbed during aeration is only 13–20% [19]. Such pronounced discrepancies in the reported growth rates and permissible limits of bicarbonate reduction—ranging from complete replacement without loss of productivity [14,15] to pronounced growth inhibition at elevated CO_2_ [17,18,19]—are apparently related not so much to differences between strains as to the fact that most studies varied only a single parameter (either the CO_2_ concentration or the NaHCO_3_ concentration) at several discrete levels, without a systematic comparison of the full gradient of bicarbonate concentrations under the same gas supply regime.

Moreover, in the studies known to us, the reduction of NaHCO_3_ concentration was investigated either at atmospheric CO_2_ levels (including an attempt to cultivate *Limnospira platensis* using exclusively atmospheric CO_2_ without bicarbonate addition [20]), or at elevated CO_2_ concentration, but it was not compared directly within a single experiment under identical cultivation conditions, which makes it difficult to separate the role of bicarbonate as a carbon source from its role as a pH buffer. We previously demonstrated the CO_2_ capture efficiency and biomass production of *Limnospira platensis* in column photobioreactors placed in a hermetic gas chamber, using a strain long adapted to elevated CO_2_ [17,21], as well as the dynamics of the carbonate–bicarbonate equilibrium in the same gas chamber [22]. The present work builds on these results by directly comparing the growth of *L. platensis* across the full gradient of NaHCO_3_ concentrations (from 0 to 100% of the reference level of 12 g/L in the modified Zarrouk medium) under a CO_2_-enriched atmosphere (3% setpoint) in the gas chamber and at atmospheric CO_2_ content on the same experimental platform.

The aim of this work is to assess the possibility of reducing, up to complete elimination, the NaHCO_3_ concentration in the nutrient medium for cultivation of *Limnospira platensis* at elevated atmospheric CO_2_ concentration in comparison with cultivation at atmospheric CO_2_ content.

To achieve this aim, the following tasks were addressed:

1. To investigate the biomass accumulation dynamics of *Limnospira platensis* at different NaHCO_3_ concentrations.
2. To study the dynamics of the pH of the nutrient medium and of the carbonate–bicarbonate equilibrium depending on the NaHCO_3_ concentration and the composition of the gas atmosphere;
3. To assess the consumption of nitrates and phosphates by the culture at different NaHCO_3_ concentrations;
4. To perform microscopic analysis of the culture and assess the proportion of live and dead trichomes for each experimental variant;
5. To determine the minimum NaHCO_3_ concentration (or the possibility of its complete elimination) at which efficient growth and high viability of the *Limnospira platensis* culture are maintained at elevated CO_2_ concentration.

## 2. Results

### 2.1. Growth

**Figure 1:**
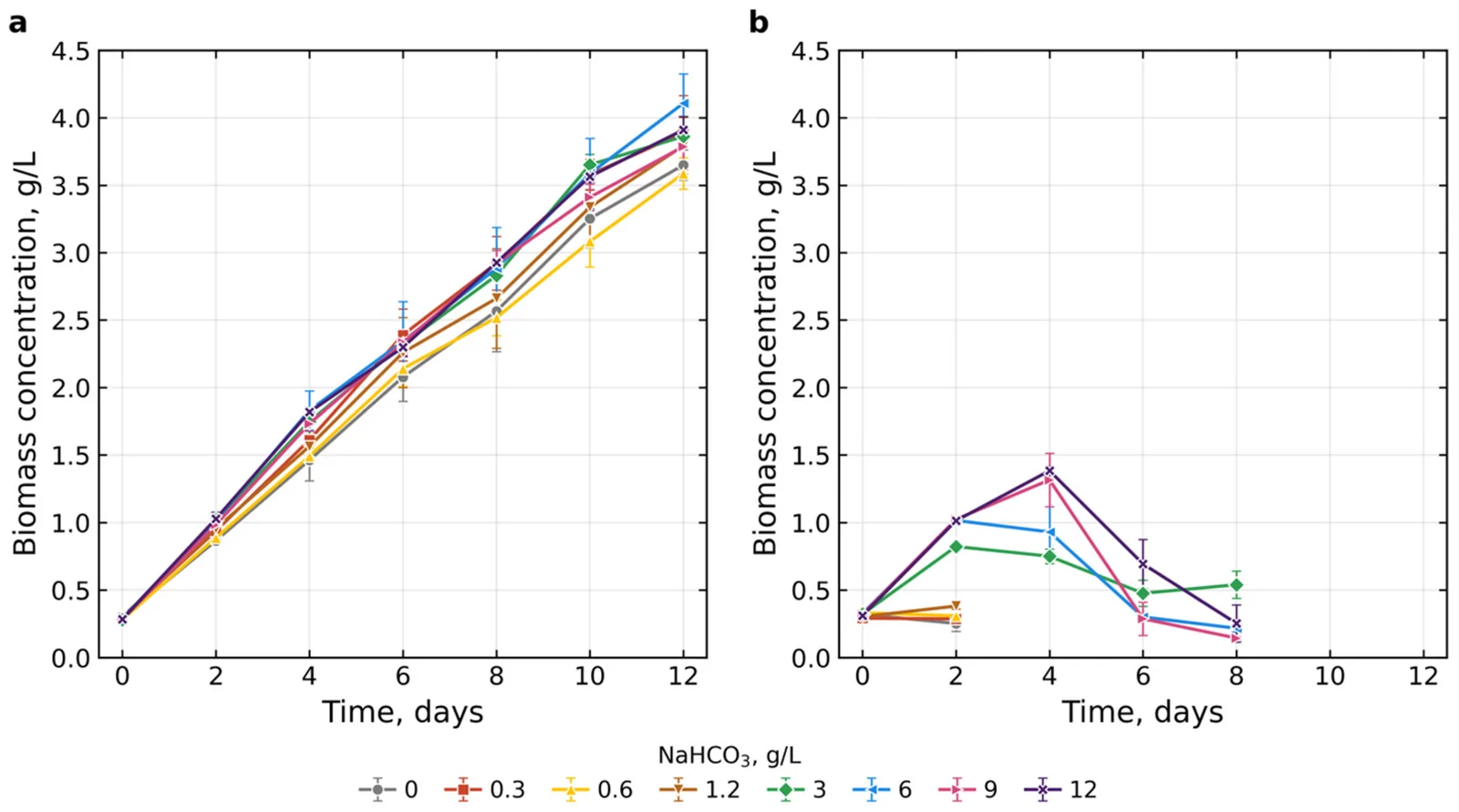
Changes in spirulina biomass concentration at different NaHCO_3_ concentrations (0–12 g/L) under 3% CO_2_ (**a**) and atmospheric (0.04%) CO_2_ (**b**). Data are presented as mean ± standard deviation (*n* = 3 for 3% CO_2_; *n* = 2 for atmospheric CO_2_). In (**b**), the curves for 0–1.2 g/L NaHCO_3_ end at day 2 and those for 3–12 g/L at day 8 because the cultures died.

**Figure A1:**
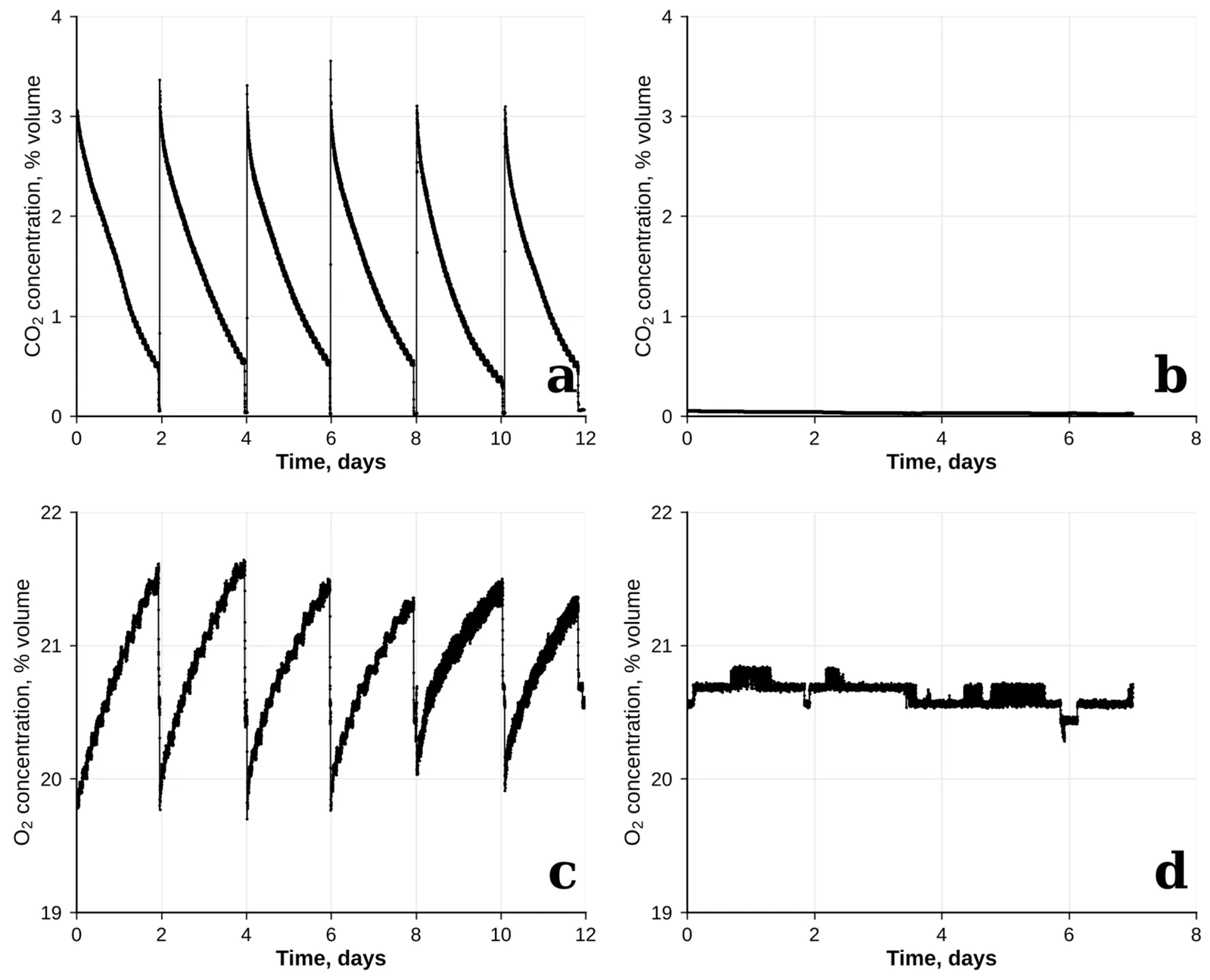
CO_2_ (**a**,**b**) and O_2_ (**c**,**d**) concentrations in the gas chamber during Experiment 1 (**a**,**c**; CO_2_ setpoint 3 vol%) and Experiment 2 (**b**,**d**; atmospheric CO_2_). Time 0 is the start of the record. In Experiment 1, the CO_2_ concentration was raised to 3.1–3.6 vol% after each sampling (days 2, 4, 6, 8 and 10) and declined to 0.4–0.5 vol% by the next one; the sharp drops are the venting of the chamber during sampling (on day 12, the chamber was vented and not refilled). The O_2_ concentration (19.6–21.7 vol%) fell at each venting and refill (return to ambient air and dilution with the added CO_2_) and rose between the refills. In Experiment 2, the CO_2_ concentration was 0.02–0.05 vol% and the O_2_ concentration 20.3–20.9 vol% throughout the record, which covers days 0–7. The readings were recorded at intervals of about 5–6 s and are shown as 1 min means; isolated spurious readings (zero values and values with a time stamp of exactly 00:00:00) were omitted.

Changes in spirulina biomass concentration at different NaHCO_3_ concentrations in the two experiments are shown in Figure 1. When spirulina was cultivated in a CO_2_-enriched atmosphere (3% setpoint; time-weighted mean ≈1.4%, Appendix A, Figure A1a), stable biomass growth was observed at all tested NaHCO_3_ concentrations (0–12 g/L) throughout the experiment. The initial biomass (0.28–0.30 g/L, day 0) did not differ significantly between variants. By day 12, the biomass reached 3.59–4.11 g/L, with the highest values recorded at 6 g/L (4.11 ± 0.22 g/L) and 12 g/L (3.91 ± 0.10 g/L) and the lowest at 0.6 g/L (3.59 ± 0.12 g/L) and 0 g/L (3.65 ± 0.11). One-way ANOVA revealed significant differences between concentrations on day 12 (F = 2.83; *p* = 0.04; Kruskal–Wallis: *p* = 0.086); however, Tukey’s post hoc test showed a significant difference only between the 0.6 and 6 g/L variants (*p* = 0.025). The effect of concentration was therefore weak but not absent: relative to the 12 g/L control (3.91 g/L), the final biomass was 0.26 g/L (6.6%) lower at 0 g/L (95% CI of the difference from −0.50 to −0.02 g/L) and 0.32 g/L (8.2%) lower at 0.6 g/L (95% CI from −0.57 to −0.07 g/L), whereas it was 0.20 g/L (5.1%) higher at 6 g/L (95% CI from −0.26 to +0.66 g/L) (Welch intervals calculated from the means and SD, *n* = 3). Thus, under 3% CO_2_ the addition of NaHCO_3_ was not required to sustain growth, although a small reduction in the final biomass in the variants with the lowest additions cannot be excluded. Growth was approximately linear (Figure 1a) and nitrate was almost completely consumed by day 12 (Section 2.3); the final biomass was therefore limited by light and nitrogen and is a conservative test of carbon supply. The productivity of the individual reactors, calculated as (B_12_ − B_0_)/12, ranged from 0.275 ± 0.009 g L^−1^ d^−1^ (0.6 g/L) to 0.318 ± 0.019 g L^−1^ d^−1^ (6 g/L) and was 0.279 ± 0.010 and 0.302 ± 0.007 g L^−1^ d^−1^ at 0 and 12 g/L, respectively (ANOVA: F = 2.66, *p* = 0.050; Kruskal–Wallis: *p* = 0.091). Regression of the day-12 biomass on the NaHCO_3_ concentration gave a slope of only 0.017 g/L per g/L of NaHCO_3_ (95% CI from −0.003 to 0.037 g/L per g/L; R^2^ = 0.12; *p* = 0.097), i.e., an increase of about 0.2 g/L (≈5%) over the whole range from 0 to 12 g/L. In an exploratory comparison the mean day-12 biomass at 0–1.2 g/L (3.73 g/L, *n* = 12) was 5% lower than at 3–12 g/L (3.92 g/L, *n* = 12; Welch’s *t*-test, *p* = 0.023). The effect was more pronounced earlier in the cultivation: relative to the control, the biomass at 0 and 0.6 g/L was lower by 16–20% on days 2 and 4, by 9–16% on days 6–10 and by 7–8% on day 12; ANOVA was significant on days 2, 4, 10 and 12 (*p* = 0.004, 0.004, 0.015 and 0.040) but not on days 6 and 8 (*p* = 0.36 and 0.14). The addition of NaHCO_3_ thus mainly accelerated the initial growth, and there was no threshold concentration below which growth failed: all tested levels, including the variant without added NaHCO_3_, supported growth.

At atmospheric CO_2_ concentration, the dynamics were different. At 0–1.2 g/L NaHCO_3_, the culture died within 2 days: on day 2 the biomass did not exceed 0.38 g/L, and no further measurements were taken. At 3–12 g/L, short-term growth was observed with a maximum on days 2–4 (0.82–1.38 g/L), after which the biomass concentration dropped sharply—by day 8 it was 0.14–0.54 g/L. These dynamics are consistent with alkalization of the medium (pH > 12), a shift in the carbonate equilibrium toward CO_3_^2−^, and complete depletion of HCO_3_^−^. Because the cultures died, Experiment 2 was monitored only until day 8, and its biomass values are OD-based estimates obtained with the same coefficient as in Experiment 1 (Section 4.6).

### 2.2. Changes in pH of Culture Medium 

**Figure 2:**
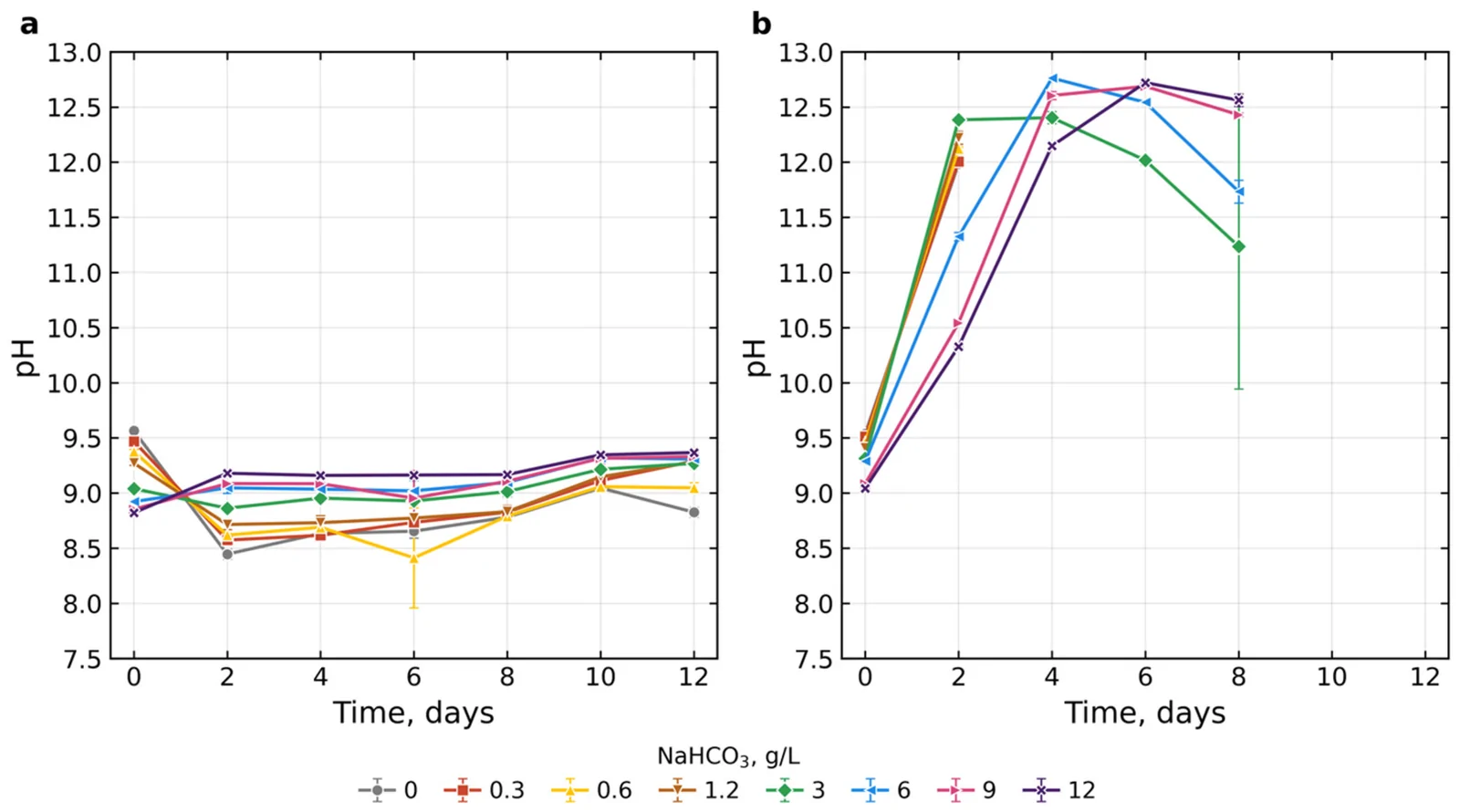
Dynamics of the pH of the spirulina culture medium at different NaHCO_3_ concentrations (0–12 g/L) under 3% CO_2_ (**a**) and atmospheric (0.04%) CO_2_ (**b**). Data are presented as mean ± SD (*n* = 3 for 3% CO_2_; *n* = 2 for atmospheric CO_2_). In (**b**), the curves for 0–1.2 g/L NaHCO_3_ end at day 2 and those for 3–12 g/L at day 8 because the cultures died; values above pH ≈ 10 are approximate.

Figure 2 shows the changes in pH during cultivation of *Limnospira platensis* with different NaHCO_3_ additions at 3% CO_2_ (a) and atmospheric (0.04%) CO_2_ (b). At 3% CO_2_, the pH of the medium remained within a narrow range of 8.40–9.40 throughout the experiment. It was noted that on day 0 the pH decreased with increasing NaHCO_3_ concentration: from 9.57 ± 0.01 at 0 g/L to 8.82 ± 0.02 at 12 g/L, reflecting the composition of the freshly prepared media (these initial differences are set by the medium preparation and were not tested statistically). By day 12, the differences between variants remained significant (F = 98.3; *p* < 0.001). The lowest pH was recorded in samples without added NaHCO_3_ (8.83 ± 0.04), and the highest at 12 g/L (9.37 ± 0.02). Relative to day 0, the pH therefore decreased by 0.74 units at 0 g/L and increased by 0.55 units at 12 g/L, i.e., the pH values of the variants converged toward 8.8–9.4 during cultivation. According to Tukey’s test, samples with bicarbonate contents of 0, 0.6, and 3 g/L differed significantly both from the other samples and from each other.

At atmospheric carbon dioxide concentration, the picture was different: as early as day 2 the pH rose to 10.3–12.4, and by days 4–8 it reached 12.75. At 0–1.2 g/L the culture died on day 2, and at 3–12 g/L by day 8. The pH values above ≈10 were measured beyond the upper calibration point of the electrode (pH 9.18) and, owing to the alkaline error of glass electrodes in sodium-rich solutions, should be regarded as approximate (Section 4.5.2).

### 2.3. Dynamics of Phosphate and Nitrate Content

**Figure 3:**
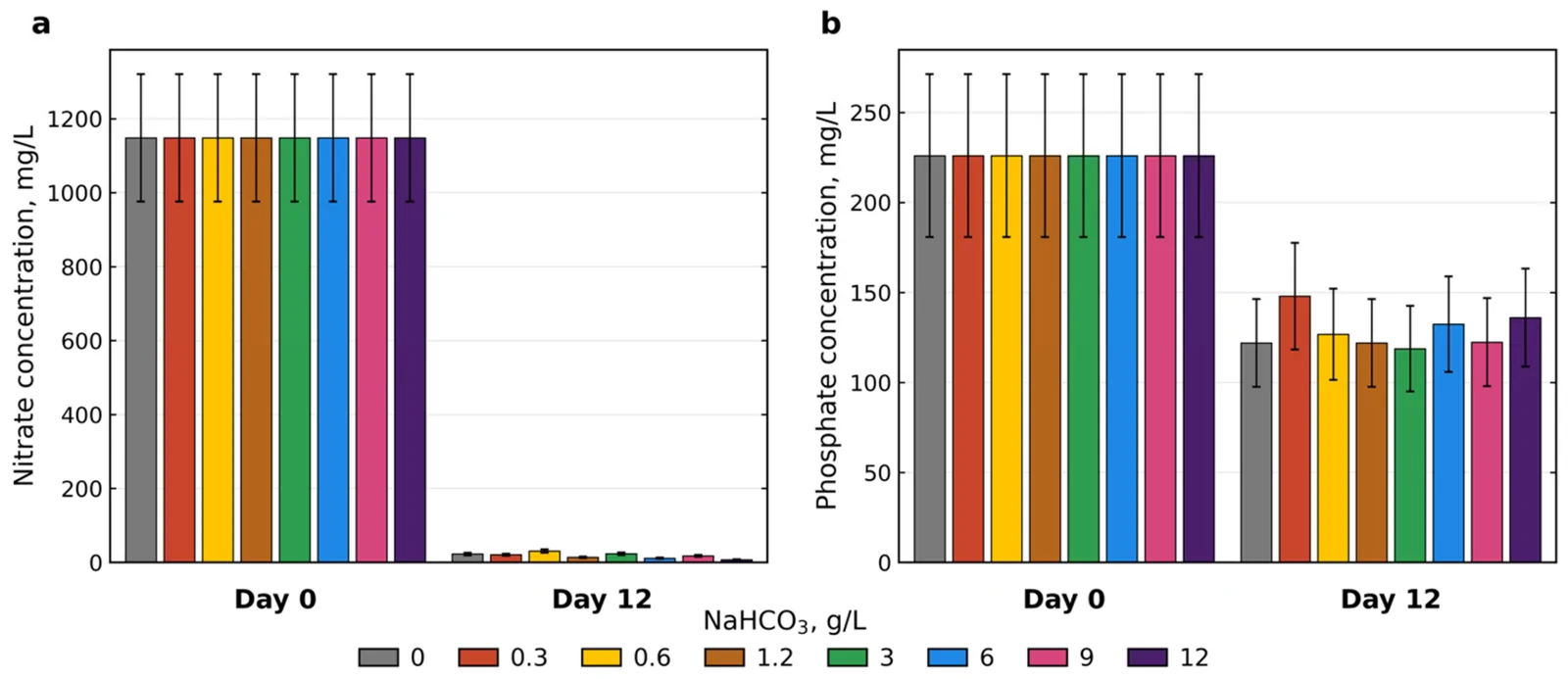
Concentrations of nitrates (**a**) and phosphates (**b**) in the medium on days 0 and 12 of cultivation of *Limnospira platensis* at different NaHCO_3_ concentrations (0–12 g/L) under 3% CO_2_. All bars are colored according to the NaHCO_3_ concentration (g/L) as shown in the common legend. Error bars correspond to the analytical error of the method (±15% for NO_3_^−^, ±20% for PO_4_^3−^), *n* = 1 for each concentration on day 12. On day 12, nitrate and phosphate were determined in one reactor per NaHCO_3_ concentration (the first reactor of each triplicate).

Figure 3a shows the change in nitrate concentration during Experiment 1 at different NaHCO_3_ concentrations. At 3% CO_2_, the nitrate concentration in the medium decreased from 1148 ± 172 mg/L (day 0) (the value calculated from the medium composition, 3.0 g/L KNO_3_, is 1840 mg/L; the lower measured value probably reflects incomplete recovery in the Mg-reduction/Griess method, so the nitrate data are used for relative comparison only) to 7.4–30.8 mg/L by day 12, depending on the NaHCO_3_ concentration. Such complete nitrate consumption (more than 97%) indicates active nitrogen metabolism of the culture throughout the experiment. The lowest residual concentrations were observed at 12 g/L NaHCO_3_, whereas at 0.6 g/L they were the highest. These differences lie within the methodological uncertainty and do not allow us to claim a significant effect of bicarbonate concentration on final nitrate consumption—in all variants, nitrogen was almost completely depleted.

Figure 3b presents the results of phosphate measurements on days 0 and 12 of Experiment 1 (at 3% CO_2_). The phosphate concentration decreased from 226 ± 45 mg/L (day 0) (275 mg/L calculated from the medium composition, i.e., within the analytical error) to 119–148 mg/L by day 12, i.e., consumption amounted to 35–47% of the initial level. The minimum residual content was recorded at 3 g/L NaHCO_3_ and the maximum at 6 g/L. As with nitrates, the differences between concentrations are comparable to the analytical error of the method (±20%) and do not allow a significant effect of NaHCO_3_ on phosphorus consumption to be identified. It is important to note that, unlike nitrate, phosphate was not exhausted—more than half of the initial amount remained in the medium at the end of the experiment.

Under atmospheric conditions, residual nitrates and phosphates could not be measured because the culture died within 8 days.

### 2.4. Changes in Carbonates and Bicarbonates

**Figure 4:**
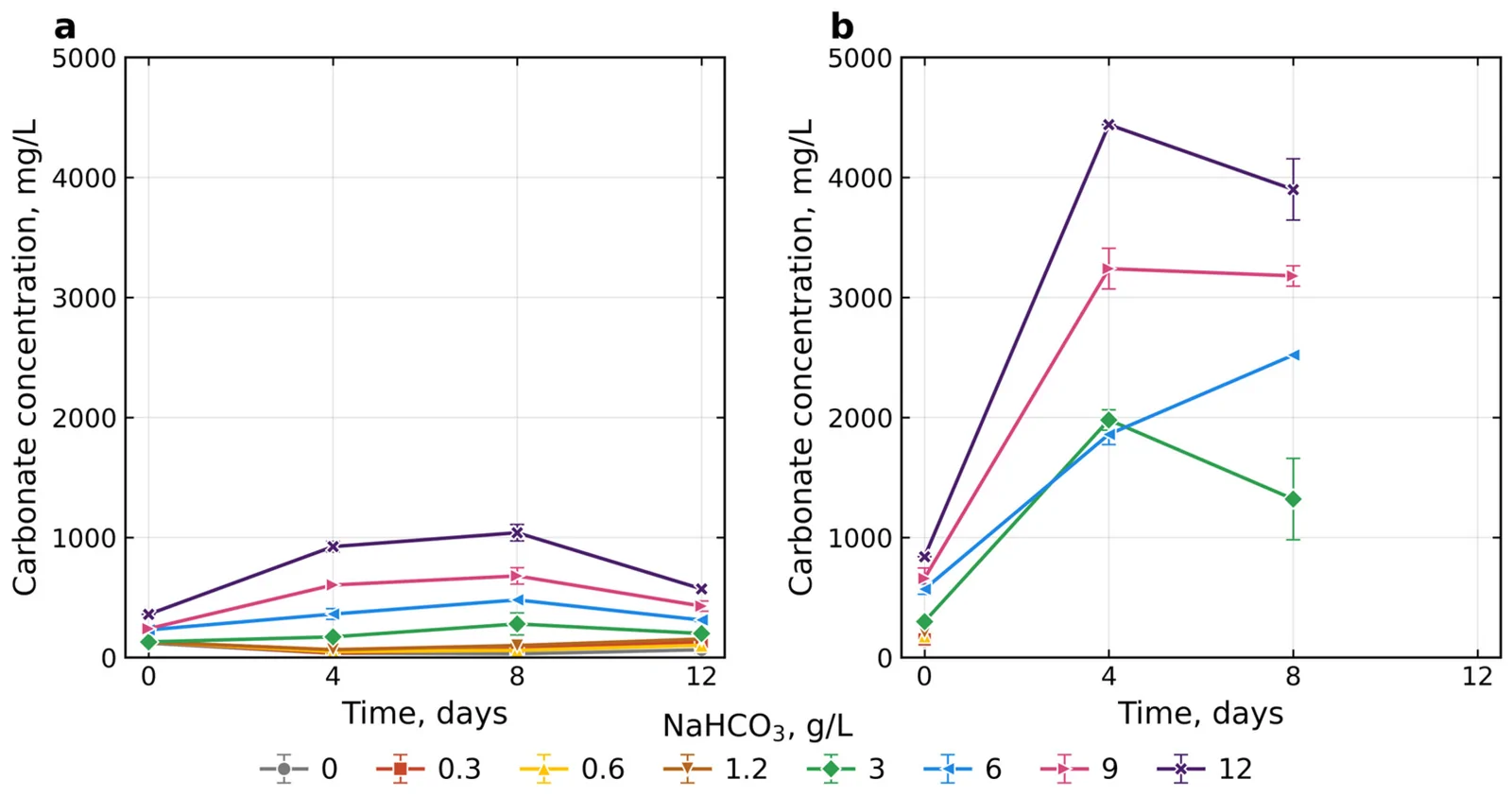
Dynamics of carbonate concentration in the medium during cultivation of *Limnospira platensis* at different NaHCO_3_ concentrations (0–12 g/L) under 3% CO_2_ (**a**) and atmospheric (0.04%) CO_2_ (**b**). Data are presented as mean ± SD (*n* = 3 for 3% CO_2_; *n* = 2 for atmospheric CO_2_). In (**b**), no measurements were made after day 0 at 0–1.2 g/L NaHCO_3_ because of culture death, and the values for days 4–8 include hydroxide alkalinity (see text).

Changes in carbonate concentration are shown in Figure 4. Under 3% CO_2_, the carbonate dynamics were non-monotonic: after a decrease by day 4, their content increased by day 8, reaching a maximum at 12 g/L (1040 ± 69 mg/L), and decreased again by day 12. The final values remained dependent on the initial NaHCO_3_ concentration. The differences between concentrations were significant on all days (ANOVA, *p* < 0.001) with homogeneous variances (Levene’s test, *p* > 0.05). On day 12, Tukey’s test distinguished four groups: a minimum at 0 and 0.6 g/L (65 and 102 mg/L), intermediate values at 0.3, 1.2, and 3 g/L (132–200 mg/L), then 6 g/L (312 mg/L) and 9 g/L (429 mg/L), and a maximum at 12 g/L (572 mg/L). For reference, the day-12 carbonate concentrations correspond to 1.1–9.5 mmol/L (from 0 to 12 g/L NaHCO_3_).

Under atmospheric conditions, the calculated carbonate concentrations increased markedly by day 4, reaching 1980 ± 85, 1860 ± 85, 3240 ± 170, and 4440 mg/L at initial NaHCO_3_ concentrations of 3, 6, 9, and 12 g/L, respectively (Figure 4b). By day 8, the corresponding concentrations were 1320 ± 339, 2520, 3180 ± 85, and 3900 ± 255 mg/L. Measurements were not available after day 0 for the 0–1.2 g/L treatments because the cultures died, and no day-12 data were available for any treatment for the same reason.

On day 4, the recalculated carbonate concentrations at 6, 9, and 12 g/L NaHCO_3_ were 32, 54, and 74 mmol/L (1.9, 3.2, and 4.4 g/L), respectively. These estimates were broadly consistent with the maximum carbonate concentrations theoretically obtainable through complete conversion of the added HCO_3_^−^ to CO_3_^2−^ (35.7, 53.6, and 71.4 mmol/L, respectively). The corresponding estimated OH^−^ concentrations were 35, 20, and 11 mmol/L, equivalent to calculated pH values of approximately 12.5, 12.3, and 12.0, compared with measured values of 12.76, 12.60, and 12.15.

The calculation for the 3 g/L NaHCO_3_ treatment on day 4 was inconsistent: the estimated carbonate concentration (34 mmol/L) exceeded the theoretical maximum of 17.9 mmol/L, while total alkalinity (67 meq/L) was approximately 20 meq/L higher than on day 0 (47 meq/L). In addition, the calculated OH^−^ concentration was approximately zero, which was inconsistent with the measured pH of 12.4. The cause of these discrepancies could not be established. Accordingly, although Figure 4b presents carbonate concentrations corrected for the contribution of OH^−^ alkalinity, the values for the 3 g/L treatment should be interpreted cautiously.

**Figure 5:**
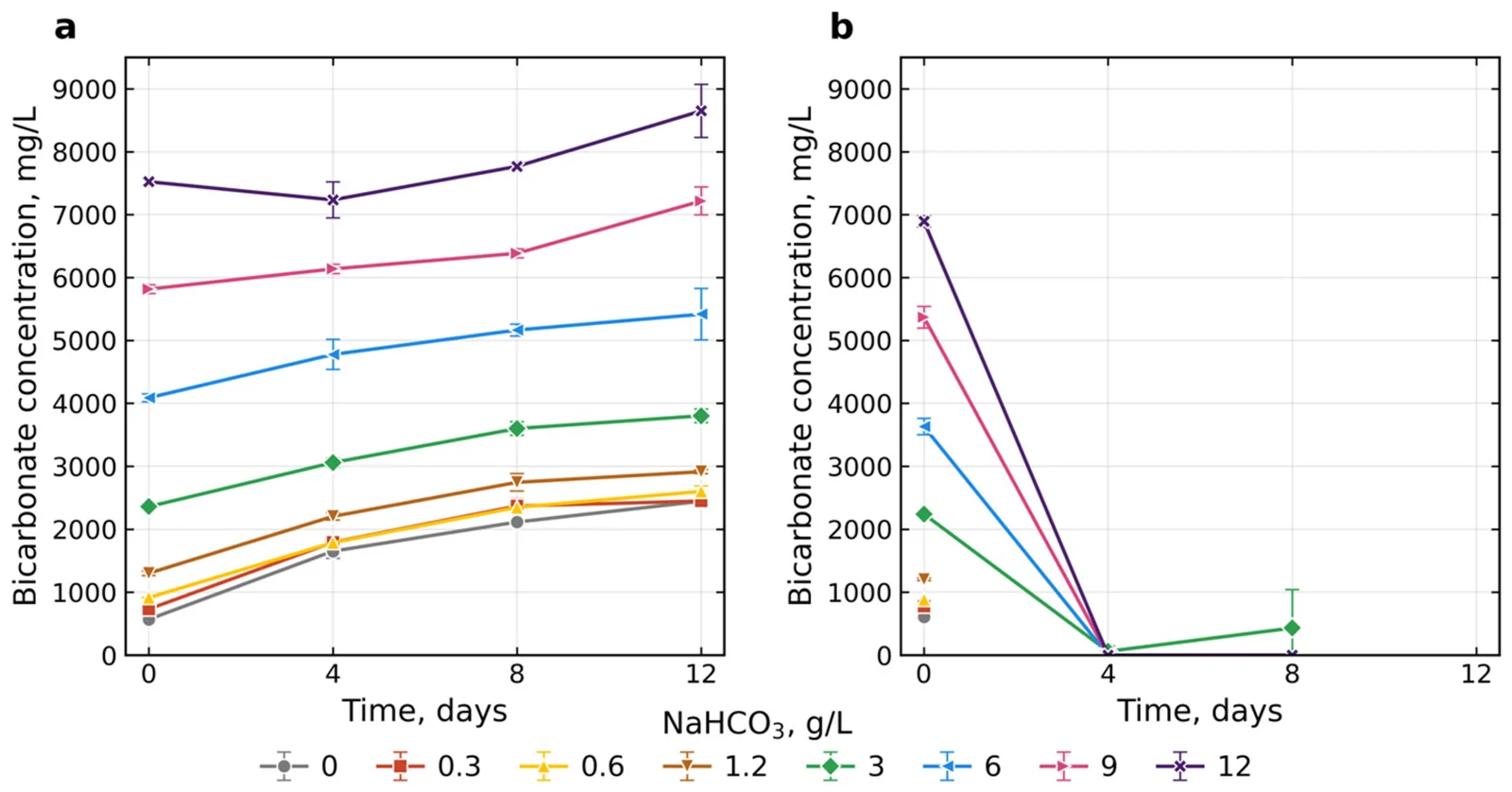
Dynamics of bicarbonate concentration in the medium during cultivation of *Limnospira platensis* at different NaHCO_3_ concentrations (0–12 g/L) under 3% CO_2_ (**a**) and atmospheric (0.04%) CO_2_ (**b**). Data are presented as mean ± SD (*n* = 3 for 3% CO_2_; *n* = 2 for atmospheric CO_2_). In (**b**), no measurements were made after day 0 at 0–1.2 g/L NaHCO_3_ because of culture death; the values for days 4–8 are approximate (Section 2.4).

The dynamics of bicarbonate ion concentration are shown in Figure 5. At 3% carbon dioxide, the bicarbonate content increased monotonically throughout the experiment in all variants. By day 12, the values reached 2445–8650 mg/L (40.1–141.8 mmol/L) depending on the NaHCO_3_ concentration: the minimum at 0–1.2 g/L (2445–2914 mg/L, not statistically different from each other), an intermediate value at 3 g/L (3802 ± 107 mg/L), then 6 g/L (5417 ± 410 mg/L) and 9 g/L (7216 ± 221 mg/L), and the maximum at 12 g/L (8650 ± 421 mg/L). The differences between concentrations were significant on all days (ANOVA, *p* < 0.001) with homogeneous variances (Levene’s test, *p* > 0.18). Tukey’s test showed that on day 12 each of the high concentrations (3, 6, 9, and 12 g/L) forms a separate statistical group, whereas at low NaHCO_3_ concentrations (0–1.2 g/L) there were no statistically significant differences by the end of the experiment.

Under atmospheric conditions, bicarbonate had almost completely disappeared by day 4. At 6, 9, and 12 g/L its content dropped to zero, and at 3 g/L trace amounts remained. For the 0–1.2 g/L variants, no measurements were taken after day 0 because of culture death; data for day 12 are absent at all NaHCO_3_ concentrations for the same reason.

### 2.5. Viability of Limnospira platensis

**Figure 6:**
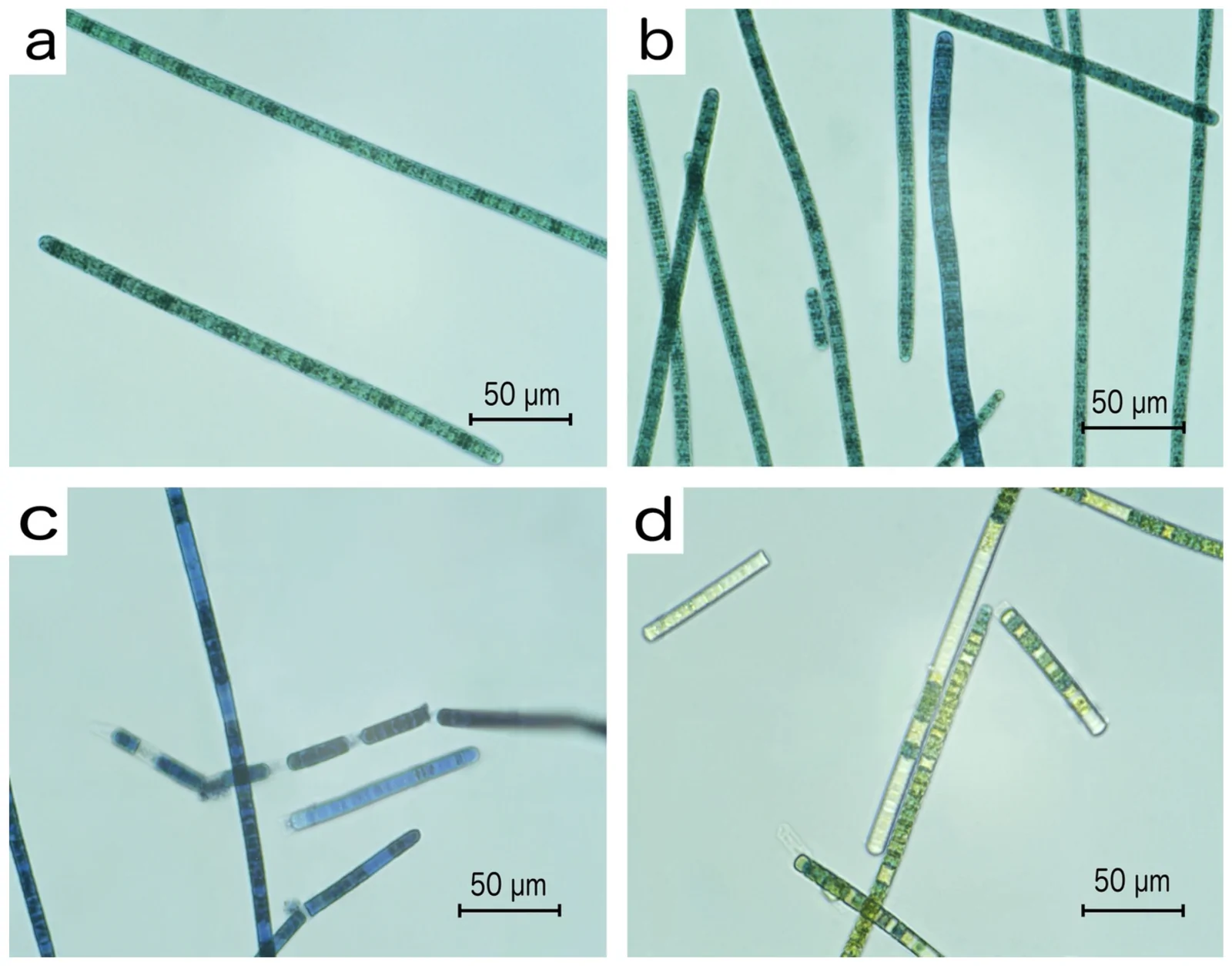
Micrographs of the *Limnospira platensis* culture: (**a**)—general view of the culture on the first day of Experiment 1, stained with methylene blue; (**b**)—culture on day 12 at a NaHCO_3_ concentration of 3 g/L under 3% CO_2_, stained with methylene blue; (**c**)—culture on day 6 at 3 g/L NaHCO_3_ under atmospheric conditions, stained with methylene blue; (**d**)—culture on day 6 at 3 g/L NaHCO_3_ under atmospheric conditions, without methylene blue staining.

Micrographs of the *Limnospira platensis* culture grown at different NaHCO_3_ concentrations are shown in Figure 6. In the 3% CO_2_ experiment, the trichomes retained their typical morphology throughout the cultivation period; they remained intact, and the proportion of damaged forms was negligible (Figure 6b). Under atmospheric conditions, on day 6 voids and breaks were found in the trichomes, indicating cell lysis and filament degradation (Figure 6c,d); no intact viable trichomes were found. Methylene blue staining confirmed these observations: at 3% CO_2_ the dye accumulated only in isolated trichomes, whereas in the experiment with atmospheric CO_2_ concentration all trichomes were stained.

**Figure 7:**
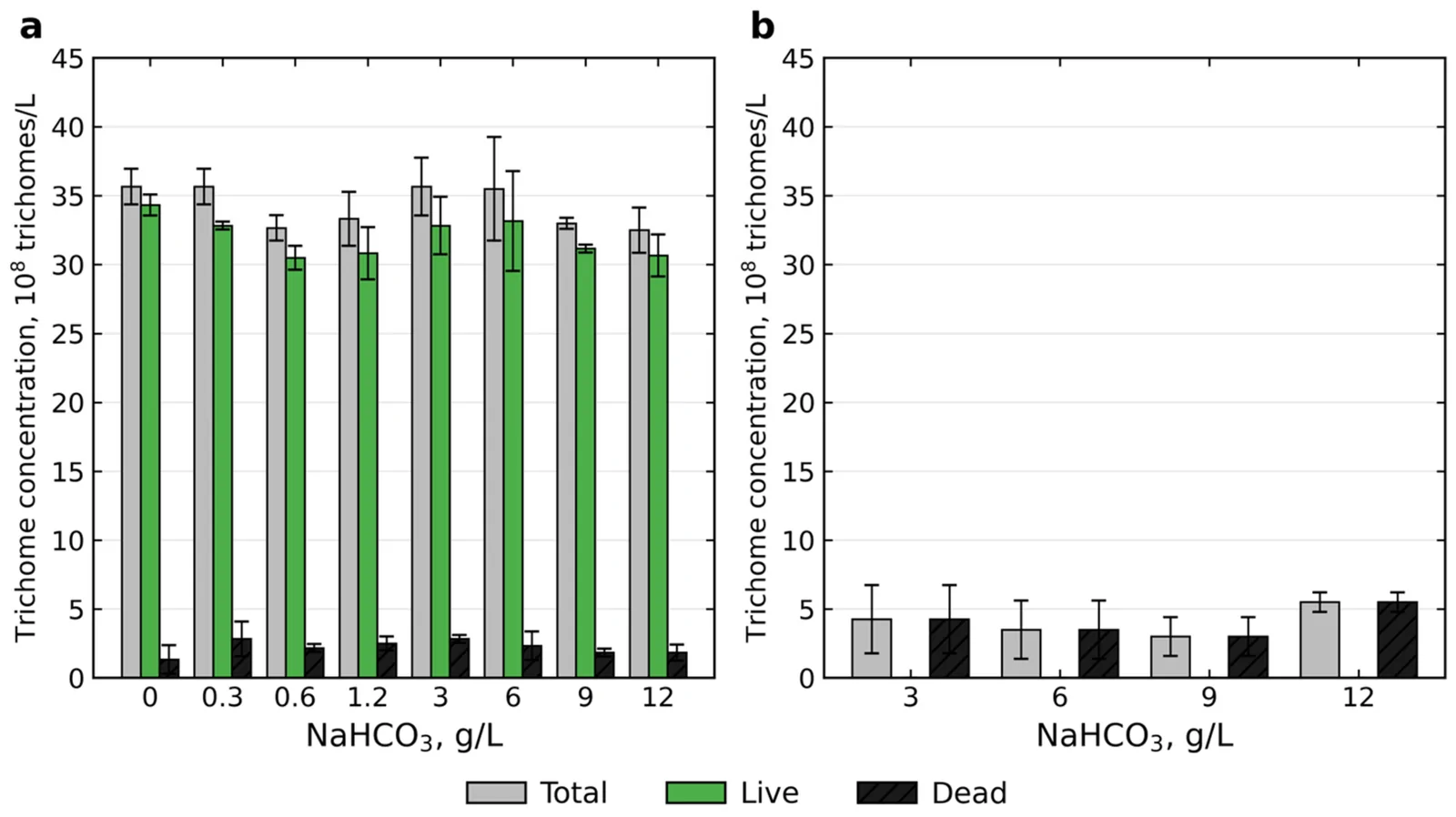
Concentration of live and dead trichomes of *Limnospira platensis* at different NaHCO_3_ concentrations (0–12 g/L): (**a**)—day 12 under 3% CO_2_ (*n* = 3); (**b**)—day 6 under atmospheric conditions (*n* = 2, surviving concentrations only). Bars show the mean, error bars show the standard deviation.

By day 12 in the 3% CO_2_ experiment, the culture retained high viability at all NaHCO_3_ concentrations (Figure 7a). The density of live trichomes ranged from 30.5 ± 0.9 to 34.3 ± 0.8 × 10^8^/L, and that of dead trichomes from 1.3 ± 1.0 to 2.8 ± 1.3 × 10^8^/L. One-way ANOVA revealed no significant differences between NaHCO_3_ concentrations in either live (F = 1.97; *p* = 0.124) or dead trichomes (F = 1.44; *p* = 0.256); the Kruskal–Wallis test gave the same results (*p* = 0.174 and *p* = 0.294, respectively). The proportion of live trichomes was 92.1–96.3% and also did not differ between variants (ANOVA: F = 1.25; *p* = 0.332; Kruskal–Wallis: *p* = 0.331). Thus, by the end of the experiment, none of the tested NaHCO_3_ concentrations had a significant effect on trichome viability—in all variants the culture remained in good physiological condition.

In the atmospheric experiment (Figure 7b), no live trichomes were found by day 6 at any of the remaining NaHCO_3_ concentrations (3, 6, 9, and 12 g/L). The density of dead trichomes was 2.0–6.0 × 10^8^/L, and viability was 0% in all variants. For the 0–1.2 g/L variants, no measurements were taken because the culture had died at an earlier time point.

## 3. Discussion

The results obtained demonstrate that the key factor determining the growth of *Limnospira platensis* in our experiments is not so much the form or amount of inorganic carbon supplied as the ability of the system to maintain the pH within a physiologically acceptable range. Under 3% CO_2_, the culture grew across the entire range of tested NaHCO_3_ concentrations, and the effect of concentration on final biomass was weak, although statistically significant. This is consistent with the view that *L. platensis* can assimilate both gaseous CO_2_ and bicarbonate [23]. In cyanobacteria, inorganic carbon is taken up mainly as HCO_3_^−^ by Na^+^-dependent and ATP-dependent transporters and as CO_2_ by NDH-1-based uptake systems, and is accumulated in the cell as HCO_3_^−^ before fixation in carboxysomes; the high-affinity systems are induced under inorganic carbon limitation and down-regulated at high CO_2_ [24,25]. In our cultures, the pH of 8.8–9.4 means that most of the dissolved inorganic carbon was HCO_3_^−^ formed from the CO_2_ absorbed from the gas phase, so our data do not allow the contributions of CO_2_ and HCO_3_^−^ uptake to be separated. It is also known that the bicarbonate form of carbon is generally assimilated by spirulina better than gaseous CO_2_ [16]; our results do not contradict this, since in the 3% CO_2_ experiment bicarbonate remained available (its pool was replenished from the gas phase), but the initial addition of NaHCO_3_ was not necessary for growth.

Under 3% CO_2_, the pH of the medium was maintained in the range of 8.40–9.40, which corresponds to the optimal values for *L. platensis*. This is explained by the fact that when the gas mixture is sparged through the medium, the absorbed CO_2_ dissolves and dissociates stepwise according to the carbonate system [9]:

CO_2_(g) ⇌ CO_2_(aq) + H_2_O ⇌ H_2_CO_3_ ⇌ H^+^ + HCO_3_^−^ ⇌ 2H^+^ + CO_3_^2−^

The supply of CO_2_ from the gas phase (raised to ≈3% after every sampling and declining to ≈0.5% by the next one; Appendix A, Figure A1a) shifts this equilibrium to the left, maintaining a sufficient H^+^ concentration and preventing the alkalization caused by photosynthetic consumption of HCO_3_^−^. The buffer system provided by the HCO_3_^−^/CO_3_^2−^ equilibrium worked stably, and the dynamics of carbonates and bicarbonates were typical of an actively growing culture: the HCO_3_^−^ content increased monotonically in all variants, reaching 2445–8650 mg/L by day 12, whereas carbonates first decreased, then rose by day 8 and fell again by day 12. We previously showed that such dynamics of the carbonate–bicarbonate equilibrium are characteristic of *L. platensis* in the same gas chamber [22]. The present study builds on these observations by demonstrating that a similar pattern of dynamics is maintained under CO_2_ enrichment (3% setpoint) over a wide range of NaHCO_3_ concentrations.

The measured pH values must be interpreted together with the gas record (Appendix A, Figure A1a). The CO_2_ concentration in the chamber was not constant: it was raised to 3.1–3.6 vol% after each refill and declined, driven by biological uptake together with gas–liquid mass transfer and mixing dynamics, to 0.4–0.5 vol% by the next sampling (≈1.3 vol% per day); the time-weighted mean was 1.4 vol%, and the concentration was above 2 vol% for only 22% of the time. The “3%” is therefore the setpoint and the peak value, not the exposure. For a solution in equilibrium with 3% CO_2_ at 27 °C ([CO_2_(aq)] ≈ 1 mmol/L), the HCO_3_^−^ concentrations found on day 12 (40–142 mmol/L) would correspond to a pH of about 7.8–8.5, whereas 8.83–9.37 was measured. The CO_2_ partial pressure calculated from the measured pH and HCO_3_^−^ on day 12 is only about 0.4 vol% (or less when ionic-strength effects are taken into account), which is close to the 0.4–0.5 vol% recorded in the chamber immediately before each sampling. The cultures were thus close to equilibrium with the chamber air at the end of each cycle but not with the 3% reached after the refills: during the cycle, photosynthetic uptake and gas–liquid mass transfer kept the dissolved CO_2_ below the level corresponding to the peak concentration. Because the pH was measured only at the samplings, the contributions of the declining gas-phase concentration and of mass-transfer limitation cannot be separated, and the scheme above describes the direction of the processes rather than the state of the system.

A simple carbon balance shows where the carbon came from. In the control (12 g/L NaHCO_3_), the medium contained 143 mmol/L of inorganic carbon at the start, about equal to the carbon fixed in the biomass by day 12 (3.91 g/L × ~47% C ≈ 153 mmol C/L; the carbon content of the biomass was assumed, not measured). However, the HCO_3_^−^ + CO_3_^2−^ pool did not decrease; it was 151 mmol/L on day 12. Practically all of the ≈160 mmol C/L incorporated into the biomass and accumulated as dissolved inorganic carbon therefore came from the gas phase, and the added NaHCO_3_ was not consumed. In the variant without added NaHCO_3_, the biomass (3.65 g/L, ≈143 mmol C/L) and the increase in the HCO_3_^−^ + CO_3_^2−^ pool from ≈9 to ≈41 mmol/L (≈175 mmol C/L in total) were likewise supplied by CO_2_ from the gas phase. This corresponds to ≈1.6–1.7 mol C per 10 L reactor over 12 days, or about 17–18% of the CO_2_ passed through the sparger (1 L/min at the time-weighted mean CO_2_ concentration of 1.4 vol% ≈ 9.6 mol per reactor; ≈21 mol if the chamber had been held at 3%), which is within the 13–20% absorption efficiency reported for sparged systems [19]; the unabsorbed gas returned to the chamber atmosphere.

The alkalinity balance is equally informative. In the variant without added NaHCO_3_, the alkalinity (HCO_3_^−^ + 2CO_3_^2−^) rose by about 25–30 meq/L during cultivation, which matches the alkalinity released by assimilation of nitrate (one equivalent per mole of NO_3_^−^; 29.7 mmol/L from 3.0 g/L KNO_3_, more than 97% of which was consumed). The K^+^ left behind after nitrate uptake is balanced by HCO_3_^−^ formed from the absorbed CO_2_. The “bicarbonate-free” variants were thus not free of bicarbonate: in addition to ≈9 mmol/L (≈0.55 g/L) of HCO_3_^−^ present at the start, introduced with the inoculum (700 mL per reactor, i.e., 7% of the working volume, grown in Zarrouk medium with 12 g/L NaHCO_3_; this corresponds to ≈0.8 g/L NaHCO_3_, or ≈10 mmol/L, carried over into every reactor), bicarbonate was generated in situ from gas-phase CO_2_ and nitrate-derived alkalinity. Our conclusion therefore applies to media in which nitrate is the nitrogen source. With ammonium, alkalinity would be consumed instead, whereas with urea, assimilation is roughly alkalinity-neutral and would not generate the alkalinity observed with nitrate. The conclusion cannot be extended to bicarbonate-free conditions in the strict sense.

A fundamentally different picture under atmospheric conditions is explained by the fact that, without an external supply of CO_2_, the equilibrium shifts to the right. Photosynthetic consumption of HCO_3_^−^ and nitrate assimilation release OH^−^ ions, while sparging with air strips CO_2_ from the medium:

HCO_3_^−^ → CO_2_ + OH^−^ (in the cell); CO_3_^2−^ + H_2_O → HCO_3_^−^ + OH^−^

These OH^−^ ions are not neutralized because the dissolution of CO_2_ from the atmosphere (CO_2_ content ~0.04%) is too slow to compensate for their formation. Growth of *Arthrospira* (*Limnospira*) is strongly inhibited at a pH of about 11.5, and near pH 12 cultures lose photosynthetic activity and bleach [26,27]. In our experiments, the *L. platensis* culture died when the apparent pH exceeded about 11–12; the mechanism of high-pH injury has not been established, and inhibition of photosystem II and carbon limitation have been proposed [28]. In our atmospheric experiments, the pH reached 12 as early as days 2–4, which is consistent with the death of the culture at low NaHCO_3_ concentrations and the short-term growth followed by degradation at 3–12 g/L. Adding bicarbonate under these conditions only delayed the moment of depletion but did not prevent alkalization. The death of the culture in air cannot be attributed to pH alone, and our experiment does not identify its cause unambiguously. It probably resulted from a combination of factors, including high pH and carbon starvation. Under our continuous high-light conditions (225 µmol m^−2^ s^−1^), rapid photosynthesis accelerated CO_2_ depletion and pH rise. While our biomass productivity (0.28–0.32 g L^−1^ d^−1^) is higher than values reported in [29] (0.04–0.09 g L^−1^ d^−1^), this difference primarily reflects our much higher light intensity (~20-fold), not necessarily an intrinsically higher growth rate of the strain. In addition, the inoculum had been adapted for six months to 3% CO_2_ and was transferred directly to air under continuous light (225 µmol m^−2^ s^−1^); cells acclimated to high CO_2_ usually down-regulate their inducible high-affinity inorganic carbon uptake, so that carbon starvation and photodamage may have set in before the CO_2_-concentrating mechanism was re-induced [24,25]. Neither an air-adapted inoculum nor the full 16.8 g/L Zarrouk medium was included as a control, and spirulina is routinely cultivated for weeks under air sparging. The result for atmospheric CO_2_ therefore applies only to our system (CO_2_-adapted inoculum, continuous light, high productivity, 1 L/min sparging), and a control with an air-adapted inoculum should be tested.

Microscopic analysis was consistent with these observations. In the 3% CO_2_ experiment, the trichomes retained their typical morphology throughout the cultivation period, and the proportion of live trichomes was 92–96% regardless of the NaHCO_3_ concentration. Under atmospheric conditions, by day 6, multiple voids and breaks appeared in the trichomes, indicating cell lysis, and viability was 0%. Methylene blue is less established as a viability stain for cyanobacteria than for yeast, so the live/dead counts are taken here as an indicator of the loss of membrane integrity; Fv/Fm or chlorophyll autofluorescence should be used as complementary methods in future work.

Thus, our results show that with CO_2_ enrichment of the chamber atmosphere (3% setpoint), *L. platensis* can grow without added NaHCO_3_—reducing the added NaHCO_3_ down to zero lowered the final biomass by no more than about 8% and did not affect culture viability. This indicates that the carbon incorporated into the biomass was supplied from the gas phase, with the bicarbonate pool formed in situ, whereas the added bicarbonate contributed mainly initial alkalinity and buffering.

Lowering the NaHCO_3_ concentration from 12 to 0 g/L also reduced the Na^+^ concentration from ≈160 to ≈17 mmol/L (NaCl only) and the ionic strength of the medium from ≈0.22 to ≈0.08 mol/L (calculated from the medium composition). Because the main HCO_3_^−^ transporters of cyanobacteria (BicA and SbtA) are Na^+^-dependent [24] and Na^+^/H^+^ exchange contributes to pH homeostasis, part of the treatment effect could reflect Na^+^ rather than carbon supply or buffering. We cannot exclude that the small decrease in the final biomass at 0–0.6 g/L reflects this; Na^+^-balanced controls (NaHCO_3_ replaced by NaCl) were not included and should be used in future work.

The result obtained at 3% CO_2_ agrees with the studies in which NaHCO_3_ was fully replaced by CO_2_ and NaOH [14] or omitted at 0.5% CO_2_ [15], and extends them to a full gradient of NaHCO_3_ concentrations examined in a single experiment. The small maximum we observed at an intermediate addition (6 g/L, 4.11 g/L) resembles the optimum at half of the standard concentration (8.4 g/L) found in [16], but in our data, it did not differ significantly from the control, so we do not claim an optimum. The 3% CO_2_ used here is below the levels that caused cell death and decreased lipid and protein contents (9% [17]) or reduced the specific growth rate (10% [18]); whether media without added NaHCO_3_ tolerate higher CO_2_ levels remains to be tested. Finally, the low CO_2_ absorption efficiency of sparging reported in [19] (13–20%) is consistent with the mass-transfer limitation found here, and previous research has demonstrated that cultivation costs can be reduced by reformulating the culture medium using less expensive chemical inputs [8]. However, such savings relate to the cost of the culture medium and do not account for the additional costs associated with CO_2_ supply.

The saving of NaHCO_3_ has to be set against the CO_2_ that must be supplied. Per kilogram of biomass produced without added NaHCO_3_ (3.65 g/L), 3.3 kg of NaHCO_3_ (12 g/L ÷ 3.65 g/L) is not needed, whereas ≈1.7 kg of CO_2_ is fixed (at ~47% C), which corresponds to ≈9–13 kg of CO_2_ supplied at the 13–20% absorption efficiency typical of sparged systems [19] if the unabsorbed gas is not recycled. In our closed chamber the unabsorbed gas was returned to the chamber atmosphere, and the losses were limited to venting and leakage. According to the analyzer record (Appendix A, Figure A1a), CO_2_ was added to the chamber six times (the initial fill and five refills, each raising the concentration by 3.0–3.6 vol%), i.e., ≈95 mol (≈4.2 kg) of CO_2_ for ≈0.87 kg of biomass formed in the 24 reactors, or at least ≈4.8 kg of CO_2_ per kilogram of biomass (assuming a free gas volume of 12 m^3^, 27 °C, minor losses due to venting during refills, and a background leak rate of 0.05% per day, Section 4.5.6), of which only about 36% was fixed in the biomass; the actual consumption was higher. The decline in the chamber CO_2_ between the refills (≈1.3 vol% per day, ≈76 mol over 12 days) was larger than the CO_2_ taken up by the cultures (≈34 mol C in the biomass at 47% C plus ≈6 mol in the increase in dissolved inorganic carbon) and the assumed leak (≈3 mol), so that about 33 mol (≈43%) of the decline remains unexplained; leakage alone would have to be ≈0.6 vol% per day, about eleven times the assumed value, to close the balance. The O_2_ rise between the refills (≈0.7 vol% per day, ≈3.4 mol per day) is consistent with the biomass estimate (≈2.8 mol C per day); the cause of the discrepancy was not established, and it does not affect the main conclusions. Moreover, bicarbonate accumulated in all variants (Figure 5a), i.e., NaHCO_3_ is not consumed under CO_2_ enrichment but is lost only when the medium is discarded; with medium recycling [12] the saving would therefore be smaller than for a once-through medium. We did not carry out a cost analysis (CO_2_ source and price, pH control, sodium supply), and the reduction in medium costs should be understood as a potential, not a demonstrated, benefit.

The robustness of the approach outside the chamber also has limits. Even under CO_2_ enrichment the variants with low NaHCO_3_ had a pH of 8.40–8.45 (Figure 2a), just below the optimum range cited in the Introduction (8.5–10.5), and at constant alkalinity a five-fold increase in the CO_2_ partial pressure (e.g., flue gas) lowers the pH by about 0.7 units, so that media with low bicarbonate may be acidified at higher CO_2_ levels. A high alkalinity also protects open cultures against contaminants, and in our earlier study [17] a shift toward green picoplankton was observed at 9% CO_2_; the risk of contamination in media with low alkalinity should be evaluated. Chlorophyll a, carotenoid, protein and phycocyanin content of the biomass grown without added NaHCO_3_ was not determined; the product quality therefore has to be examined before the approach is applied.

This study has several limitations. (i) The CO_2_ treatment was not replicated: the two atmospheres were studied in two successive experiments, so that CO_2_ is confounded with the experimental run, and the comparison between them is descriptive. (ii) The inoculum was adapted to 3% CO_2_ and transferred directly to air; no air-adapted inoculum and no full-strength (16.8 g/L) Zarrouk control was included. (iii) Experiment 2 had two replicates per variant, and nitrate, phosphate on day 12 were measured in one reactor per variant; the reactors were arranged in order of NaHCO_3_ concentration, without randomization. (iv) Biomass was calculated from OD with a single coefficient, which may be less accurate when nitrogen is exhausted or the culture is dying. (v) pH values above 9.18 and the titration-based speciation are approximate: at pH > 11 the titration includes hydroxide, and the CO_3_^2−^/HCO_3_^−^ ratios found at pH ≈ 9.4 were lower than expected from the second dissociation constant. (vi) Only one batch cycle without subculturing was tested. (vii) The variant without added NaHCO_3_ contained ≈0.8 g/L of NaHCO_3_ carried over with the inoculum, and the CO_2_ consumption of the chamber was only roughly estimated from the analyzer record. (viii) The CO_2_ concentration was not constant: it was raised to ≈3% after each sampling and declined to ≈0.5% by the next one (time-weighted mean ≈1.4%), so that “3% CO_2_” denotes the setpoint and the peak value; the cultures were exposed to a saw-tooth CO_2_ regime, and the results cannot be extrapolated to a constant CO_2_ level. The results should therefore be regarded as a proof of principle.

## 4. Materials and Methods

### 4.1. Experimental Setup

**Figure 8:**
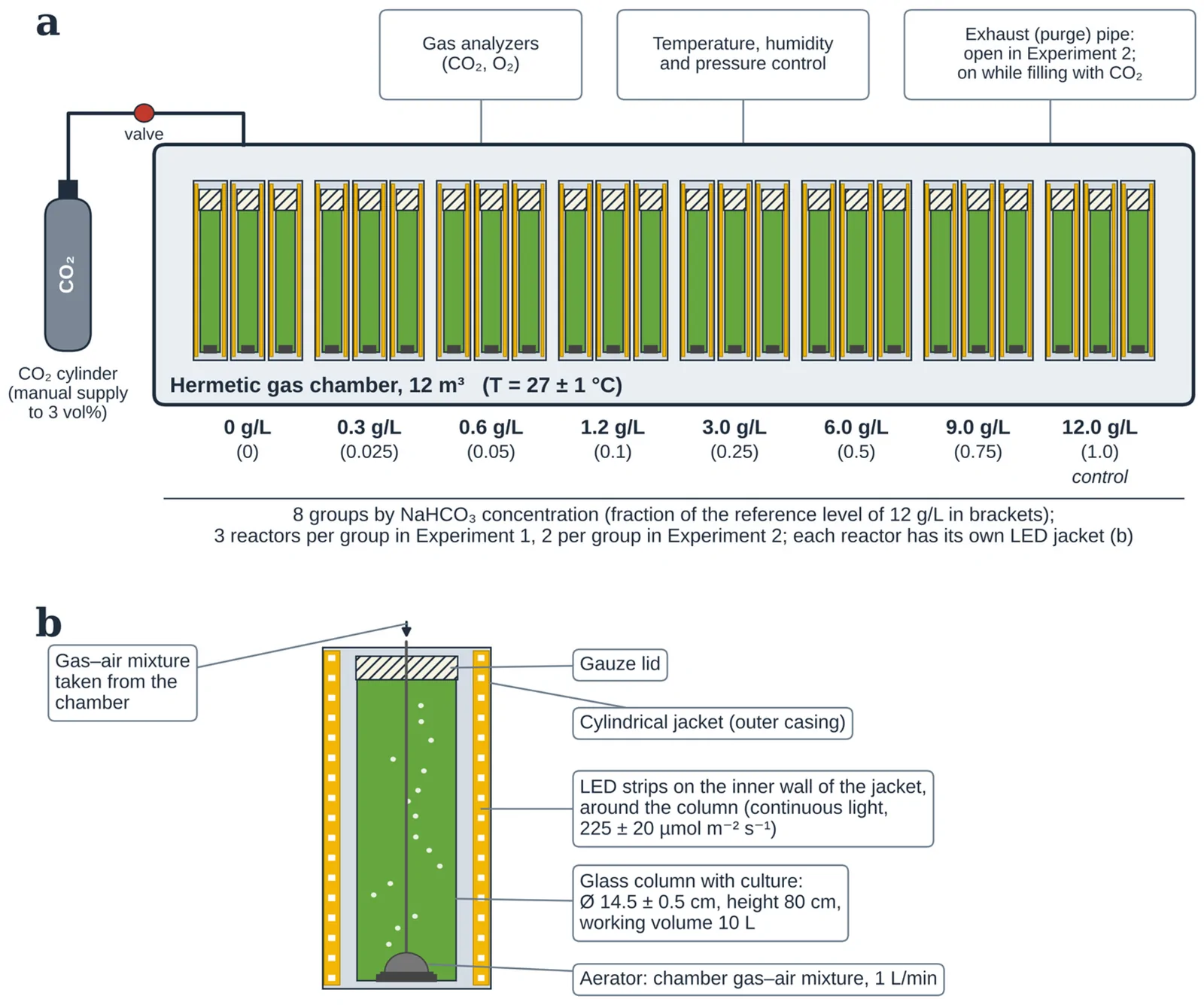
Experimental setup for cultivation of *Limnospira platensis* in a gas chamber: (**a**) general layout of photobioreactors by NaHCO_3_ concentration in the nutrient medium (3 reactors per group in Experiment 1, 2 reactors per group in Experiment 2); (**b**) close-up of a single photobioreactor with its circular illumination system. Schematic, not to scale. Image generated by AI.

The experiments were carried out in a hermetic atmospheric gas chamber with a volume of 12 m^3^, equipped with temperature, humidity, and pressure control systems, as well as a multichannel gas analyzer for continuous recording of O_2_ and CO_2_ concentrations. The chamber was connected to CO_2_ cylinders, which made it possible to maintain the specified atmospheric composition. Cylindrical glass photobioreactors (PBRs) with a volume of 10 L each (height 80 cm, inner diameter 14.5 ± 0.5 cm; the volume of 10 L is the working volume), covered with gauze lids, were placed on the floor inside the chamber. Sparging of the gas–air mixture taken directly from the chamber atmosphere was carried out through aerators located at the bottom of each PBR at a flow rate of 1 L/min per reactor; the unabsorbed gas returned to the chamber atmosphere. Illumination of all photobioreactors was continuous (24 h/day), provided by LED strips, and set at the same intensity level for all reactors, equal to 225 ± 20 µmol m^−2^ s^−1^. Lighting was provided by FITOLUX-A144-10mm 24V Warm3000-Red LED strips (SMD 2835, 144 LEDs/m, 3000 K + 660 nm, CRI > 90). The LED strips were arranged around the photobioreactors in such a way that all columns were illuminated equally from all sides. The light intensity was measured on the inner surface of an empty column using an LM-12 lux meter (EKSIS, Moscow, Russia; measurement error ≤ ±8%) and expressed as photosynthetic photon flux density (PPFD) using a spectrum-specific conversion factor of 0.0315 µmol m^−2^ s^−1^ per lux. Continuous light was used to ensure uninterrupted growth of the biomass. The temperature in the photobioreactors was maintained at 27 ± 1 °C. The CO_2_ concentration in the chamber (setpoint 3 vol%) was controlled manually: CO_2_ was fed into the chamber through a pipe by opening the gas cylinder, the exhaust pipe was switched on to prevent a pressure rise, the CO_2_ content (vol%) was measured by the gas analyzers, and the supply was stopped as soon as 3% was reached. Because the cultures took up CO_2_, its concentration declined between the refills (Appendix A, Figure A1a), so that “3% CO_2_” denotes the setpoint and the peak value after refilling, not a constant level. An illustration of the experimental setup is shown in Figure 8.

### 4.2. Experimental Design

To study the effect of reducing the concentration of sodium bicarbonate (baking soda, NaHCO_3_) in the nutrient medium on the growth rate of *Limnospira platensis* under different gas atmosphere compositions, two sequential experiments were carried out (Experiment 1 lasted 12 days; Experiment 2 was monitored until day 8, when all cultures had died), differing only in the composition of the gas chamber atmosphere:

Experiment 1: The chamber atmosphere was enriched with carbon dioxide to a concentration of 3 vol%;

Experiment 2: The hermetic chamber door remained closed while the purge (ventilation) pipe was open, which provided free gas exchange with the surrounding environment and maintained the atmospheric CO_2_ level in the chamber (~0.04 vol%; analyzer readings 0.02–0.05 vol%, Appendix A, Figure A1b) throughout the experiment. The two experiments were carried out one after the other; the CO_2_ factor is therefore not replicated and is confounded with the experimental run so that the comparison between the atmospheres is descriptive and no statistical test between them was applied.

Within each of the two experiments, the photobioreactors were divided into eight groups differing in the initial NaHCO_3_ concentration in the nutrient medium: 0; 0.3; 0.6; 1.2; 3.0; 6.0; 9.0, and 12.0 g/L, corresponding to 0; 0.025; 0.05; 0.1; 0.25; 0.5; 0.75, and 1.0 of the reference NaHCO_3_ concentration in the modified Zarrouk medium (12 g/L, which is 71% of the standard 16.8 g/L; see Section 4.3). This concentration is that of the medium chosen by the producers of the inoculum (Lomonosov Moscow State University), to which the strain had been adapted. The group with a concentration of 12.0 g/L (1.0 of the reference level) was considered the control. Each concentration was represented by three (Experiment 1) or two (Experiment 2) independent replicate photobioreactors (each inoculated and sampled separately), so Experiment 1 included 8 × 3 = 24 photobioreactors and Experiment 2 included 8 × 2 = 16 photobioreactors. Only two reactors per variant were used in Experiment 2, because without CO_2_ enrichment and with low NaHCO_3_ the cultures were expected to die, and this experiment was intended only as a demonstration of the day on which the culture dies at different NaHCO_3_ concentrations. The reactors were placed in the chamber in order of increasing NaHCO_3_ concentration (Figure 8); the positions were not randomized.

All photobioreactors within one experiment were filled with *Limnospira platensis* inoculum with the same preset initial optical density of the microalgal suspension. The inoculum volume was 700 mL per reactor (7% of the working volume), and the inoculum was grown in Zarrouk medium containing 12 g/L NaHCO_3_; it therefore carried over ≈0.8 g/L of NaHCO_3_ into every reactor, which explains the HCO_3_^−^ concentration of about 0.55 g/L found at day 0 in the variant without added NaHCO_3_ (Figure 5a).

Figure 8 was AI-generated (Claude Sonnet 5.5, Anthropic, San Francisco, CA, USA) and verified by the authors.

### 4.3. Strain and Nutrient Medium

In this work, we used a *Limnospira platensis* strain long adapted (for 6 months) to an elevated CO_2_ concentration (3 vol%) in the atmosphere. The strain *Arthrospira (Limnospira) platensis* rsemsu P Bios was obtained from the collection of the Laboratory of Renewable Energy Sources, Lomonosov Moscow State University (Moscow, Russia). For the adaptation, the culture was transferred directly (in one step, without a gradual increase in CO_2_) to a 3 vol% CO_2_ atmosphere in a modified Zarrouk medium containing 12 g/L NaHCO_3_, under the same light intensity as in this study (225 µmol m^−2^ s^−1^), and was subcultured every two weeks during the six months of adaptation. Cultivation was carried out in modified Zarrouk medium of the following composition: NaHCO_3_—variable concentration (see Section 4.2) instead of the standard 16.8 g/L; KNO_3_ 3.0 g/L; K_2_HPO_4_·3H_2_O 0.66 g/L; K_2_SO_4_ 1.0 g/L; MgSO_4_·7H_2_O 0.2 g/L; NaCl 1.0 g/L; CaCl_2_ 0.04 g/L; FeSO_4_·7H_2_O 0.018 g/L; EDTA 0.08 g/L; trace elements for Zarrouk medium 1 mL/L. The medium was prepared with distilled water.

### 4.4. Cultivation Procedure

Experiment 1 lasted 12 days; Experiment 2 was monitored until day 8, when all cultures had died. After the photobioreactors were filled with the inoculum, the chamber was hermetically closed and filled with the required gas mixture (see Section 4.2), after which the gas concentrations inside the chamber were monitored continuously. Every two days the gas mixture was vented from the chamber and the chamber was opened—always at the same time of day—after which suspension samples were taken from the photobioreactors; then the chamber was resealed and refilled to the target atmospheric composition. According to the gas analyzer records (Appendix A, Figure A1), in Experiment 1 the CO_2_ supply was interrupted at each sampling for 30–120 min (median ≈85 min) between the start of venting and the refill; after the refill, the concentration rose to ≥2.8 vol% within 5 min (peaks of 3.1–3.6 vol%) and then declined to 0.4–0.5 vol% by the next sampling (≈1.3 vol% per day; time-weighted mean over the experiment 1.4 vol%). At each sampling, 50 mL of suspension was taken from each reactor and was not replaced with fresh medium.

The optical density (OD) and pH of the suspension were determined every two days (on days 0, 2, 4, 6, 8, 10, and 12) for all photobioreactors. On days 0, 4, 8, and 12, the concentrations of carbon-containing components of the nutrient medium—carbonate (CO_3_^2−^) and bicarbonate ions (HCO_3_^−^)—were additionally determined. On days 0 and 12 of the experiment, the concentrations of nitrates and phosphates in the nutrient medium were additionally determined, the state of the culture was monitored microscopically.

The temperature sensors and the gas analyzer recorded continuously (every 5–6 s; Section 4.5.6), and the readings were saved at least once every 7 days throughout the experiment. The CO_2_ and O_2_ records of both experiments are given in Appendix A (Figure A1).

### 4.5. Analytical Methods

#### 4.5.1. Optical Density

The OD of the suspension was measured on an Expert-003 spectrophotometer (Econix-Expert, Moscow, Russia) at a wavelength of 655 nm in cuvettes with an optical path length of 5 mm.

#### 4.5.2. pH Determination 

The pH of the nutrient medium was measured with an Expert-pH pH meter (Econix-Expert, Moscow, Russia) with automatic temperature compensation; the instrument was calibrated every 7 days using buffer solutions with pH 4.01, 6.86, and 9.18. The measurement error was ±0.01 pH units. Readings above pH 9.18, the upper calibration point, are extrapolations and, owing to the alkaline (sodium) error of glass electrodes, are approximate; the stated error applies within the calibration range only.

#### 4.5.3. Determination of Nitrate and Phosphate

Nitrate concentration was determined photometrically at a wavelength of 525 nm after reduction of nitrates to nitrites with metallic magnesium, followed by reaction of the resulting nitrites with Griess reagent.

Phosphate concentration was determined by the molybdate method on an Expert-003 spectrophotometer (Econix-Expert, Moscow, Russia) at a wavelength of 700 nm; the method is based on the formation of phosphomolybdic heteropoly acid and its subsequent reduction to a blue compound whose color intensity is proportional to the orthophosphate content.

#### 4.5.4. Determination of Carbonates and Bicarbonates

The concentrations of carbonate and bicarbonate ions were determined by direct titration with hydrochloric acid, with the equivalence points identified from the change in pH (Expert-pH pH meter (Moscow, Russia)). The samples were filtered through filter paper and titrated on the day of sampling; aliquots of 3–30 mL (depending on the expected alkalinity) were diluted with distilled water to about 20 mL and titrated with 0.1 M HCl (0.01 M HCl was used for the first end point in some series, see below). The volume of acid to the first equivalence point (pH ≈ 8.3), V_1_, corresponds to the conversion of CO_3_^2−^ to HCO_3_^−^, and the total volume to the second equivalence point (pH ≈ 4.5), V_2_, to the titration of all carbonate species; the concentrations were calculated as

[CO_3_^2−^] = c(HCl)·V_1_/V_s_ (1)

and

[HCO_3_^−^] = c(HCl)·(V_2_ − 2V_1_)/Vs (2)

where V_s_ is the sample volume (molar masses of 60 and 61 g/mol were used to convert to mg/L; when the first end point was titrated with 0.01 M HCl and the titration was continued with 0.1 M HCl, the amounts of acid c·V were summed accordingly).

The carbonate concentrations obtained in the experiment with atmospheric (0.04%) CO_2_ were calculated from titration measurements with the contribution of OH^−^ alkalinity accounted for. Because the cultures reached pH values of approximately 11–12, the concentration of HCO_3_^−^ was considered negligible. Under these conditions, the alkalinity measured at the first titration endpoint (pH 8.3) includes contributions from both OH^−^ and CO_3_^2−^; therefore, the hydroxide contribution was subtracted to estimate the carbonate concentration. The calculated values consequently represent the estimated CO_3_^2−^ concentration rather than the combined OH^−^ and CO_3_^2−^ alkalinity.

#### 4.5.5. Microscopic Analysis

Microscopic examination of the culture was performed using a Levenhuk MED Series light microscope (Levenhuk, Inc., Tampa, FL, USA) at ×100 and ×400 magnification. Cell viability was assessed by differential staining with methylene blue, based on the different membrane permeability of live and dead cells. Trichomes that remained green (unstained) were counted as live and trichomes stained blue as dead. Trichomes were counted if they lay entirely within the square or crossed its upper or right boundary; trichomes crossing only the lower or left boundary were not counted. Live and dead trichomes were counted in a Goryaev chamber in 10 large squares, followed by recalculation per 1 mL of suspension taking the chamber volume into account (the values in Section 2.5 are expressed per 1 L).

#### 4.5.6. Composition of the Chamber Gas Atmosphere

The composition of the chamber gas atmosphere (O_2_, CO_2_) was recorded continuously with a multichannel gas analyzer at a measurement interval of 5–6 s. The records are shown in Appendix A (Figure A1).

The background leak rate of the chamber (0.05 vol% per day) was taken from our earlier work [21], in which it was determined in a cell-free control run in the same 12 m^3^ chamber: a photobioreactor filled with 70 L of Zarrouk medium but not inoculated with microalgae was placed in the chamber, and the mean decline in the CO_2_ concentration, which includes dissolution in the medium and diffusion through leaks, was ≈2 × 10^−3^ vol% per hour. The leak rate was not re-measured in the present study and is used in Section 3 as an assumed value.

### 4.6. Calculated Quantities

The dry biomass concentration of spirulina in the suspension was calculated using the formula:

C_spirulina_ = OD_655_ × k (3)

where k is an empirical conversion coefficient (g/L per OD unit) determined from direct weighing of dry biomass after filtration and freeze-drying on day 12 of Experiment 1. In this work, k = 0.85 g L^−1^ per OD unit was applied to all variants and to both experiments. Because Experiment 2 had no day 12 (the cultures had died), and the OD of dying, bleached or nitrogen-depleted cultures does not necessarily follow dry weight, the biomass concentrations in these cases are OD-based estimates. Suspensions were diluted as necessary so that the OD measured fell within the working range of the instrument (0.3–0.6), and the dilution factor was taken into account in the calculation.

### 4.7. Statistical Analysis

Statistical analysis was performed in Python 3.13.13 using pandas 3.0.3, NumPy 2.4.6, SciPy 1.17.1, statsmodels 0.14.6, Matplotlib 3.10.9, and Seaborn 0.13.2. Data are presented as mean ± standard deviation (SD) unless otherwise stated. The significance level was set at *p* < 0.05.

For each parameter (biomass, pH, carbonates, bicarbonates, trichome count), the normality of the distribution within each group was tested using the Shapiro–Wilk test (which has little power with *n* = 3 per group, so the parametric results were cross-checked with the Kruskal–Wallis test), and the homogeneity of variances using Levene’s test. Differences between NaHCO_3_ concentrations were assessed by one-way analysis of variance (ANOVA) with Tukey’s post hoc test (Tukey HSD). The Kruskal–Wallis test was used as a nonparametric confirmation; when the results of the parametric and nonparametric tests were consistent, the parametric result was reported; when they were not consistent (the day-12 biomass), both are given. For biomass, one-way ANOVA was performed only for the end point (day 12); for pH, carbonates, and bicarbonates, it was performed for each measurement day (pH: days 0, 2, 4, 6, 8, 10, and 12; carbonates and bicarbonates: days 0, 4, 8, and 12). For microscopic data (live, dead, and viable trichomes), ANOVA was performed for day 12 in the 3% CO_2_ experiment. Effect sizes at day 12 are given as differences in the means from the 12 g/L control with 95% confidence intervals (Welch’s t interval). To take the repeated measurements in the same reactor into account, each reactor was characterized by its productivity, (B_12_ − B_0_)/12, which was compared between the variants by one-way ANOVA (with the Kruskal–Wallis test as a check); the day-12 biomass was in addition regressed on the NaHCO_3_ concentration by ordinary least squares, and the biomass on each sampling day was compared between the variants by one-way ANOVA (without correction for multiple days, as an exploratory analysis).

In the atmospheric experiment, the small sample size (*n* = 2) and the death of the culture in most variants did not allow formal statistical tests to be performed; therefore, the data of this experiment are presented descriptively. Because the two atmospheres were studied in two successive experiments, the CO_2_ factor is not replicated and is confounded with the experimental run; the comparison between the experiments is therefore descriptive.

For nitrates and phosphates on day 12, only one measurement per NaHCO_3_ concentration was available (*n* = 1) because the analysis was performed for one reactor per NaHCO_3_ concentration (the first reactor of each triplicate); therefore, no statistical comparisons were made. The error bars in the corresponding figure reflect the analytical error of the spectrophotometric method (±15% for NO_3_^−^ and ± 20% for PO_4_^3−^, determined from calibration curves) rather than biological variability.

## 5. Conclusions

The study showed that under CO_2_ enrichment of the gas phase (3% setpoint, ≈1.4% on average), *Limnospira platensis* grew across the entire range of added NaHCO_3_ concentrations (0–12 g/L), with the final biomass at 0 and 0.6 g/L being 7–8% lower than in the 12 g/L control, whereas in our system (inoculum adapted to 3% CO_2_ and transferred directly to air) the culture died at all NaHCO_3_ concentrations up to 12 g/L. This indicates that, in a nitrate-based medium and over one batch cycle, the supply of CO_2_ from the gas phase rather than the initial concentration of baking soda determined whether the culture could grow. A possible reduction in medium costs remains to be demonstrated by a CO_2_ supply and cost analysis. Future research should focus on testing growth with an inoculum adapted to a soda-free medium over several subcultures, including controls with an air-adapted inoculum and with the full 16.8 g/L medium, replicated CO_2_ treatments and Na^+^-balanced (NaCl) controls; on assessing the effect of the absence of bicarbonate on the biochemical composition of the biomass (protein, carbohydrates, phycocyanin, lipids, pigments); on studying the dynamics of the carbonate–bicarbonate equilibrium in a medium without added NaHCO_3_ at various CO_2_ concentrations; and on scaling up in pilot photobioreactors with an economic assessment of eliminating bicarbonate and CO_2_ supply.

## Data Availability

The original contributions presented in the study are included in the article and in Appendix A (Figure A1: CO_2_ and O_2_ records of the chamber); per-reactor raw data are provided in Supplementary File S1. Further inquiries can be directed to the corresponding author.

## Supplementary Materials

The following supporting information can be downloaded at https://www.mdpi.com/article/10.3390/plants15213425/s1, File S1: Per-reactor raw data.

## Appendix A. Data on the CO_2_ and O_2_ Concentrations in the Gas Chamber

Figure A1 shows the CO_2_ and O_2_ concentrations recorded by the multichannel gas analyzer in the gas chamber during Experiment 1 (CO_2_ setpoint 3 vol%) and Experiment 2 (atmospheric CO_2_). In Experiment 1, the time-weighted mean CO_2_ concentration was 1.4 vol%; it was above 2 vol% for 22% of the time and below 1 vol% for 38% of the time.
